# Intervention for a correct medication list and medication use in older adults: a non-randomised feasibility study among inpatients and residents during care transitions

**DOI:** 10.1007/s11096-024-01702-4

**Published:** 2024-02-10

**Authors:** Ahmed Al Musawi, Lina Hellström, Malin Axelsson, Patrik Midlöv, Margareta Rämgård, Yuanji Cheng, Tommy Eriksson

**Affiliations:** 1 Department of Biomedical Science and Biofilm – Research Center for Biointerfaces, Faculty of Health and Society, Malmö University, Malmö, Sweden; 2 Department of Medicine and Optometry, eHealth Institute, Linnaeus University, Kalmar, Sweden; 3 Pharmaceutical Department, Region Kalmar County, Kalmar, Sweden; 4 Department of Care Science, Faculty of Health and Society, Malmö University, Malmö, Sweden; 5 Department of Clinical Sciences Malmö, Center for Primary Health Care Research, Lund University, Malmö, Sweden; 6 Department of Materials Science and Applied Mathematics, Faculty of Technology and Society, Malmo University, Malmo, Sweden

**Keywords:** Aged, Medication adherence, Medication error, Medication reconciliation, Patient discharge summary, Patient transfer

## Abstract

**Background:**

Medication discrepancies in care transitions and medication non-adherence are problematic. Few interventions consider the entire process, from the hospital to the patient’s medication use at home.

**Aim:**

In preparation for randomised controlled trials (RCTs), this study aimed (1) to investigate the feasibility of recruitment and retention of patients, and data collection to reduce medication discrepancies at discharge and improve medication adherence, and (2) to explore the outcomes of the interventions.

**Method:**

Participants were recruited from a hospital and a residential area. Hospital patients participated in a pharmacist-led intervention to establish a correct medication list upon discharge and a follow-up interview two weeks post-discharge. All participants received a person-centred adherence intervention for three to six months. Discrepancies in the medication lists, the Beliefs about Medicines Questionnaire (BMQ-S), and the Medication Adherence Report Scale (MARS-5) were assessed.

**Results:**

Of 87 asked to participate, 35 were included, and 12 completed the study. Identifying discrepancies, discussing discrepancies with physicians, and performing follow-up interviews were possible. Conducting the adherence intervention was also possible using individual health plans for medication use. Among the seven hospital patients, 24 discrepancies were found. Discharging physicians agreed that all discrepancies were errors, but only ten were corrected in the discharge information. Ten participants decreased their total BMQ-S concern scores, and seven increased their total MARS-5 scores.

**Conclusion:**

Based on this study, conducting the two RCTs separately may increase the inclusion rate. Data collection was feasible. Both interventions were feasible in many aspects but need to be optimised in upcoming RCTs.

**Supplementary Information:**

The online version contains supplementary material available at 10.1007/s11096-024-01702-4.

## Impact statements

- The correct medication list intervention can identify discrepancies and establish a correct medication list upon discharge in collaboration with patients and physicians.
- The person-centred intervention can address participants’ barriers to medication adherence, reduce concerns about medicines, and improve medication adherence.
- This study highlights the need to conduct large-scale studies (RCTs) to study the effectiveness of the interventions.

## Introduction

Several studies and systematic reviews have emphasised the clinical, economic, and humanitarian consequences for patients who do not receive optimal medication treatment [1–4]. Sub-optimal medication treatment may occur in different sections of the healthcare system. Hence, treatment issues in the form of discrepancies in, and patients’ non-adherence to, prescribed medications for chronic illnesses need to be examined in relation to the healthcare system in order to identify the sources of error. One source of medication error is care transitions, where patients are at high risk of being exposed to medication discrepancies and errors, which could lead to adverse clinical outcomes and increased healthcare costs [5–9].

According to the Swedish Patient Safety law and the National Board of Health and Welfare’s regulations, patients should get individual information about their health and care conditions, including a written discharge summary with a list of prescribed medications, namely a correct medication list (Correct-ML). However, discrepancies in discharge summaries are common in Sweden and the rest of the world. Studies have found significant error rates: up to 87% of discharged patients are affected [1, 10–17]. Thus, ensuring a Correct-ML at discharge is necessary.

Furthermore, adherence to the correct medication list is imperative. In their report on medication adherence, the World Health Organization (WHO) states that fewer than 50% of individuals with chronic illnesses take their medications as prescribed, and that” *increasing the effectiveness of adherence interventions may have a far greater impact on the health of the population than any improvement in specific medical treatments*” [18]. A systematic review and meta-analysis confirms that medication non-adherence is still a global challenge [19]. Medication adherence is a multidimensional phenomenon that may be affected by patient-related factors, such as beliefs and knowledge about medications [18].

Therefore, intervention studies examining the entire process for a correct medication list and a medication adherence intervention are needed. We have designed a project to further develop the Lund Integrated Medicines Management (LIMM) model [20], focusing on a Correct-ML at hospital discharge and a person-centred intervention aiming to improve medication adherence through randomised controlled studies. The LIMM model is a systematic approach to individualise and optimise drug treatment in elderly patients admitted to hospital. This model involves systematic activities for medication review, including medication reconciliation. This model has shown evidence for better care processes and clinical and economic outcomes [20]. However, the LIMM model has not previously been applied in a long-term perspective among patients in their home environment. Also, the model has not been studied among non-Swedish-speaking patients. Immigrants make up around 20% of the total population in Sweden, and most have Arabic as their native language [21]. However, they are often excluded from research [22]. Corresponding to the Medical Research Council’s recommendations for developing research and services in healthcare [23] and in preparation for future definitive randomised and controlled trials (RCTs), we first test, investigate, and develop tools and procedures for our Correct-ML and adherence interventions in a feasibility study.

### Aim

In preparation for planned randomised controlled trials, this study aimed (1) to investigate the feasibility of recruitment and retention of patients, and data collection for planned randomised controlled trials to reduce discharge information discrepancies and improve patient medication adherence, and (2) to explore the outcomes of the interventions.

### Ethics approval

Ethical approval was obtained from the Swedish Ethical Review Authority (2021-00255; 06-03-2021).

## Method

The reporting of this study follows the Consolidated Standards of Reporting Trials (CONSORT) extension [24]. Although the CONSORT extension was developed primarily for randomised pilot and feasibility studies, it is also the most appropriate reporting guideline for non-randomised feasibility studies [25].

### Study setting, population, and recruitment

This feasibility study was performed from October 2021 to October 2022, where participants were followed for three to six months.

Persons 60 years or older who were self-handling at least five continuous medications at home were eligible for inclusion. The study excluded persons classified as having an accepting attitude towards medications (Table 1) using the Beliefs About Medicines Questionnaire-Specific (BMQ-S) [26] and as being not forgetful using the Medication Adherence Report Scale (MARS) [27, 28].

**Table 1:** Description of questionnaires used in this study

Questionnaire	Description
Beliefs About Medicines Questionnaire-Specific (BMQ-S)	A ten-item questionnaire assessing a person’s subjective beliefs of necessity and concern about prescribed medications: five items represent the necessity sub-scale, and five represent the concern sub-scale. Each item is answered on a five-point Likert scale ranging from *strongly agree* to *strongly disagree*. Scores from individual items are summed within each sub-scale, ranging between 5 and 25, where higher scores indicate a high degree of necessity and concern. Scores from the two sub-scales can be dichotomised to give four attitude categories: sceptical (high concerns, low necessity), ambivalent (high concerns, high necessity), neutral (low concerns, low necessity), and accepting (low concerns, high necessity) [29]
Medication Adherence Report Scale (MARS-5)	A five-item questionnaire for measuring self-reported medication adherence. Each item is answered on a five-point Likert scale from *always* to *never*. Summed item scores give a total score between 5 and 25. Higher scores indicate higher adherence. Different opinions exist regarding an appropriate cut-off [30], and no gold standard exists for dichotomising MARS [31]. In this study, participants with total scores of 20 or above are considered to have high adherence

A pharmacist from the research team (research pharmacist) recruited the study population from orthopaedic and internal medicine wards at a university hospital in Southern Sweden and from a residential area. People from the residential area were recruited through a health promotion programme with activities for immigrants and through a community pharmacy. Based on the focus of the planned RCTs, this study consists of two interventions—a correct medication list intervention and a person-centred adherence intervention—since they are the focus of the planned RCTs. Hospital patients were included in both interventions, whereas the other group was included only in the adherence intervention. The hospital/community pharmacy participants were Swedish-speaking, while the others from the residential area were Arabic-speaking. All participants received verbal and written information and were included after giving written informed consent.

### Questionnaires and definitions

Table 1 describes the questionnaires used in the study. BMQ-S and MARS-5 were used to assess beliefs about medications and adherence at inclusion and follow-up. Both questionnaires have been translated into Swedish and validated: BMQ-S [26, 32, 33] and MARS-5 [27, 32, 34, 35]. *Medication discrepancy* was defined as omission or commission of medication, dosing errors, or changes in a dose frequency [8]. Definitions of terms used in the medication list process at the hospital and follow-up interview are presented in Table 2.

**Table 2:** Definitions of terms used to describe discrepancies in the medication list

Term	Definition
EHR-ML	The medication list in the electronic health record, i.e., medications that the patient receives during their hospital stay
N-ML	The National Medication List. It lists all current electronic prescriptions and pharmacy-dispensed prescriptions
Pat-use	Medications used as stated by the patient
Correct-ML	The correct medication list with medications that the patient has an indication for. This list represents what the patient used at home and considers possible changes during their hospital stay. It is established through a pharmacist’s medication reconciliation and accepted by the patient and discharging physician
Discharge-ML	The medication list in the discharge information summary that all patients are supposed to get at discharge. It should include comments on medication changes during the hospital stay

### Correct medication list intervention

**Fig. 1:**
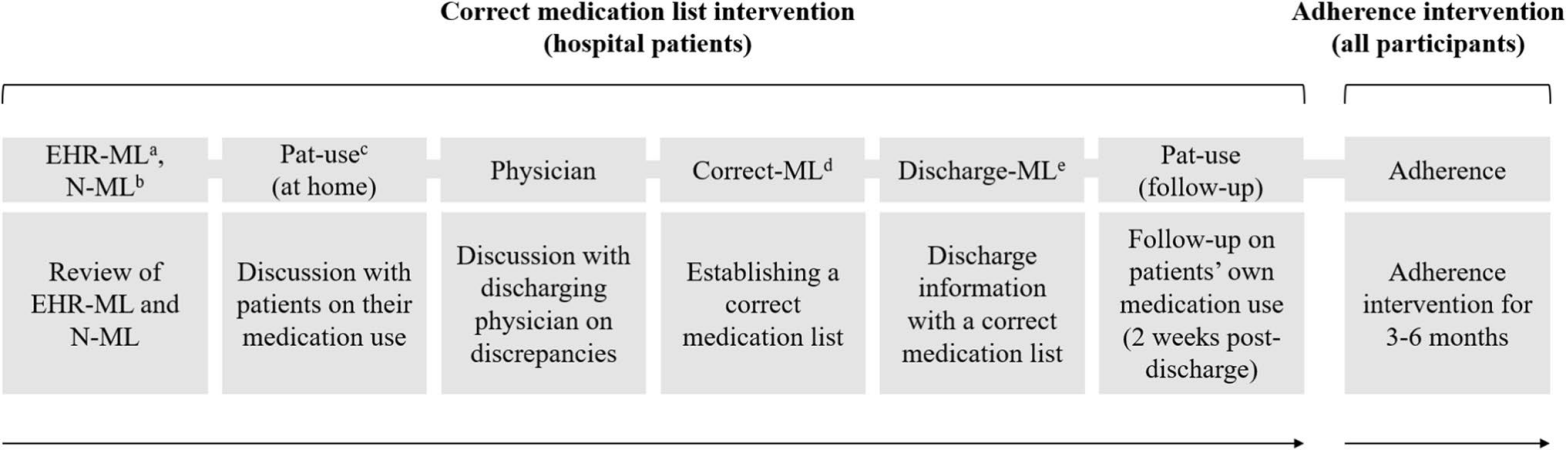
Intervention processes for a correct medication list and medication adherence in hospital and residential participants. ^a^The medication list in the electronic health record, ^b^The national medication list, ^c^Patient use, ^d^Correct medication list, ^e^Discharge medication list

All medication discrepancies, origin, and intervention results were documented for all intervention steps using a checklist. The process for establishing a Correct-ML is presented in Fig. 1. The research pharmacist conducted a medication reconciliation at inclusion using LIMM forms [20]. The medication list in the electronic health record (EHR-ML) and the national medication list (N-ML) were reviewed to discuss the patient’s medication use at home. Discrepancies were listed, discussed, and agreed upon with the responsible discharging physician, who was then able to correct the Discharge-ML based on the established Correct-ML. In turn, the pharmacist deleted the prescriptions in the N-ML that the patient should no longer use.

Two weeks after hospital discharge, a pharmacist performed a follow-up patient interview. The Pat-use was evaluated and compared to the Correct-ML established at discharge. During the follow-up interview, the pharmacist also provided the patient with information on the Correct-ML and any deviations between the Discharge-ML and the Correct-ML.

### Medication adherence intervention

The adherence intervention was initiated for participants recruited from the hospital after the correct medication list intervention was completed (Fig. 1). A person-centred approach was chosen as it considers different adherence dimensions and has shown promising results [36–38]. Since forgetfulness is also a reason for non-adherence [18], we included dispensing support as part of the adherence intervention. Based on the current medication list and patient interview, a type 2a medication review was conducted for all participants [39].

### Person-centred meetings

The adherence intervention was inspired by the Gothenburg model for person-centred care [40], comprising partnership, narrative, and documentation. Thus, the first step of this intervention involved initiating a partnership between the pharmacist and the participant during the first meeting based on the participant’s narrative about their medicines (Table S1, supplementary material). The second step was documenting the participant’s narrative, and the last step was implementing a health plan, including the participant’s long- and short-term medication-related goals. The narrative and the health plan were both documented during this first meeting.

The adherence intervention was ongoing for three to six months, during which all participants were offered at least one consultation each month. The intervention was mainly held in person in the participant’s home, but a few meetings were conducted by telephone. A follow-up on the health plan was made during each consultation, and the plan was adjusted according to each participant’s needs.

### Dispensing of medication

Two to three weeks after discharge, the participants were offered memory and dosing support using the medication dispensing device Medimi Smart (Medimi, Lund Sweden). Based on the medication list and Pat-use established at the follow-up interview, the device was programmed to send a signal, with sound and light, for each scheduled dosing time. The participants confirmed the signal and took the medications, and the device registered the dispensing time. An adherence rate of 80% meant that the signal was confirmed within two hours 80% of the time. Medimi Smart was refilled every two to four weeks when almost empty.

### Statistical tests

Basic descriptive statistics (mean and standard deviation) were computed for all variables. A paired samples t-test was used to evaluate differences in BMQ-S and MARS and medication discrepancies between EHR-ML and Discharge-ML. Due to the small sample size, the results were double-checked with the non-parametric method (Wilcoxon signed rank test), where we derived the same conclusions as the paired sample t-test. All data analyses were performed using IBM SPSS 26.

## Results

### Feasibility of recruitment and retention

A total of 87 persons were asked to participate, and 12 completed the study (Table 3). The mean (SD) of prescribed medications was 11 (4.7), the mean age was 75 years (9.6), and eight participants were female. The reasons for exclusion were refusing to participate (n = 52), withdrawing consent (n = 20), and not meeting the inclusion criteria (n = 2). One patient died during the adherence intervention period but was included where data were available. Participants were recruited within five months.

**Table 3:** Inclusion of participants for the medication list and adherence interventions

	Hospital (n)	Residential (n)
Asked	78	9
Included	7	6
Medication list intervention		–
Follow-up (2 weeks)	7	–
Adherence intervention		
Follow-up (visits, hp^a^)		
1	7	6
2	7	6
3	7	6
4	6	6
5	6	6

Residential participants were only asked to participate in the adherence intervention

^a^Health plan

### Feasibility of interventions and data collection

It was possible to collect data from the N-ML and the participants’ medication use before hospital admission to identify discrepancies in the EHR-ML. Discussing medication discrepancies with the responsible physicians, deleting old prescriptions from the N-ML, and performing follow-up interviews two weeks post-discharge were possible for all patients (Table 3). However, the discussion with physicians often occurred shortly before patient discharge, making it difficult for physicians to correct the Discharge-ML in time. Consequently, this procedure was not entirely feasible. Collecting data on BMQ-S and MARS-5 at study inclusion and termination was acceptable to the participants; all could complete both questionnaires. During the adherence intervention, the pharmacist could use the participants’ narratives and health plans to set individual goals and update the health plan during the follow-up period as intended. For each participant, three to five home visits were conducted (Table 3 and Table S2).

### Outcome of the correct medication list intervention

There were 24 medication discrepancies in the EHR-ML compared to the Correct-ML, with a mean of 3.4 (4.1) per participant (Table S3). Six of the seven hospital participants had at least one discrepancy. Omission accounted for 14 discrepancies and commission for 10. All discrepancies were communicated, discussed, and accepted as errors by the responsible discharging physicians, but only ten discrepancies were corrected in the discharge summary.

### Outcome of the medication adherence intervention

At study termination, 10 participants had decreased their total concern scores in BMQ-S compared to the scores at inclusion (Table 4). Mean necessity scores did not change at study termination compared to inclusion. Six participants (four residential participants, three of whom were Arabic-speaking, and two hospital participants) moved to the most positive attitude category in BMQ-S (i.e., accepting); all were ambivalent at inclusion. There was an increase in total MARS-5 scores at study termination compared to scores at inclusion, and seven participants increased their total scores. Of those, four were Arabic-speaking. According to MARS-5, eight participants were classified as high adherers at inclusion, compared to 11 at termination. Six participants used the Medimi Smart device, with a mean adherence rate of 97%, and all were classified as high adherers according to MARS-5.

**Table 4:** Summary of results from the adherence intervention

No	BMQ-S^a^								MARS-5^e^				Study time in weeks and (Number of visits)	Medimi-Smart, adherence (%)
	Inclusion				Termination				Inclusion		Termination			
	N^b^	C^c^	N–C dif.^d^	Attitude Group	N	C	N–C dif	Attitude Group	Points	Classification adherence	Points	Classification adherence		
1	23	15	8	Amb^f^	18	10	8	Accept^i^	19	Low	24	High	20 (4)	–
2	15	18	− 3	Scept^g^	15	18	− 3	Scept	20	High	20	High	16 (3)	97
3	17	20	− 3	Amb	14	17	− 3	Scept	23	High	25	High	20 (5)	83
4	16	23	− 7	Amb	–	–	–	–	21	High	–	–	12 (3)	97
5	22	16	6	Amb	15	7	8	Neut	24	High	24	High	16 (3)	–
6	15	13	2	Neut^h^	17	22	− 5	Amb	24	High	24	High	16 (4)	89
7	18	15	3	Amb	25	5	20	Accept	22	High	24	High	24 (5)	100
8	23	25	− 2	Amb	22	20	2	Amb	13	Low	17	Low	24 (4)	–
9	25	16	9	Amb	22	5	17	Accept	25	High	24	High	24 (4)	–
10	25	17	8	Amb	18	6	12	Accept	10	Low	24	High	16 (3)	–
11	16	22	− 6	Amb	25	13	12	Accept	19	Low	25	High	20 (4)	–
12	24	24	0	Amb	23	18	5	Amb	21	High	21	High	20 (5)	87
13	25	21	4	Amb	20	12	8	Accept	11	Low	23	High	20 (4)	–
Mean	20	20	15	–	20	12	6.7	–	19	–	23	–	19 (4)	97
SD	4.5	3.7	5.4	–	3.9	5.5	7.9	–	5	–	2.4	–	–	6.7

^a^Beliefs About Medicines Questionnaire, ^b^Necessity, ^c^Concern, ^d^Necessity-Concern differential, ^e^Mecication Adherence Report Scale-5, ^f^Ambivalent, ^g^Sceptical, ^h^Neutral, ^i^Accepting

Table S4 describes experiences from the adherence intervention and the intervention’s process and progression for one hospital participant and one residential participant.

## Discussion

Out of 87 eligible candidates, we could only recruit 13 participants within five months. Difficulties in recruiting patients to studies are common [41]. For our study, the main reason could be the complexity of the study for the participants: two interventions were tested simultaneously, involved several documents for the participants to read and complete, and included several physical follow-up visits. If the interventions are separated into two RCTs, we believe more participants would be willing to participate.

Our study suggests that the method to identify medication discrepancies is feasible. During the intervention process, all discrepancies were discussed and accepted by responsible physicians. However, not all discrepancies were corrected in the discharge medication list by the physicians, and most patients were consequently discharged with discrepancies remaining in their discharge summaries. The main reason for the remaining discrepancies was that they were discussed too late, so the physicians did not have time or missed the chance to correct them in the discharge summary. To ensure that the physician can correct the discrepancies in the Discharge-ML in the planned RCT, the discussion with the physician must be conducted well in advance of the discharge.

Although a physical and a telephone follow-up interview on the correct medication list after discharge proved feasible, the latter would be more time and cost-effective. Hence, we will choose a telephone follow-up in the planned RCT.

In our study, six of seven patients had at least one discrepancy in their medication list, with a mean of 3.4 discrepancies per patient. A previous study showed that 38% of 933 patients had at least one discrepancy, with a mean of 0.87 per patient [11], whereas another found that 87% of 200 patients had at least one discrepancy [13]. Both studies used methods for identifying and comparing discrepancies similar to those in our study; however, only a few patients in the first study received a medication reconciliation, which could explain the lower proportion of patients with discrepancies.

Most studies on medication list discrepancies lack a clear intervention. Some studies report interventions in stages of the medication process from the hospital to the home [42–44]. We found only one study focusing on all aspects of the process [45], where an RCT of 178 patients used a similar intervention to ours. Patients were called by telephone 30 days after discharge to compare self-reported medication use with the medication list in the discharge summary. Unexplained discrepancies between the discharge list and the patient’s self-reported medications were common in the control and intervention groups: 65% and 61%, respectively. However, the study does not mention that a correct medication list was established in the discharge summary. In our intervention, we actively tried to resolve discrepancies in the discharge summaries.

Furthermore, our study suggests that the adherence intervention was feasible in many aspects, but some refinements can be made. The follow-up visits in our intervention were conducted physically in the participants’ homes. However, this may have been a hindrance: it may have contributed to the low number of participants. Moreover, a systematic review and meta-analysis showed that adherence interventions were less effective when delivered in the participants’ homes than in other locations, such as pharmacies or clinics [46]. Therefore, our suggestion for the planned RCT is to make the location choice more flexible and provide a digital option.

Obtaining the participants’ narrative and establishing the health plan were both feasible, and we could use both to initiate a discussion with the participants about their medications, set goals for the intervention, and revise their health plans during the follow-up visits.

The BMQ-S and MARS-5 were feasible to use. The participants were able to complete the questionnaires at inclusion and termination. The adherence intervention yielded positive results, where six participants proceeded to the most positive attitude category in the BMQ-S (i.e., accepting); notably, three of them were Arabic-speaking. Moreover, a reduction in total concern scores in the BMQ-S was seen for 10 out of 12 participants, and all the Arabic-speaking participants had reduced concern scores. The intervention also led to an increase in total MARS scores.

The results from the BMQ-S and MARS-5 are positive and are partly in line with a previous RCT that studied adherence and beliefs about medicines in 89 patients [47]. The study included patients with sub-optimal adherence (i.e., less than 20 total scores in the MARS-5). The intervention was led by a pharmacist who met the patients twice. A simple form was used where the pharmacists and the patients set goals based on each patient’s adherence barriers and followed up on it in the next visit; thus, no specific (therapeutic/theory-based) method was used to intervene with the patients. The study showed a significant increase in total MARS scores, but in contrast to our study, there was no increase in necessity or decrease in concern in the BMQ. The reason could be that they did not include patients with high concern scores, so no significant improvements were possible.

One notable aspect of this feasibility study concerned language barriers. The hospital and residential participants who underwent the adherence intervention were either Swedish- or Arabic-speaking. However, the research pharmacist who conducted the interventions spoke both languages fluently and could acceptably navigate language, culture, and other issues. Our study indicates that Arabic-speaking patients seem to have needs that must be explored in more detail using qualitative methods. The populations included in the current study are diverse. Factors affecting medication adherence such as social, economic, cultural, health beliefs, and access to medications [18], may differ between the populations in our study, making comparison difficult. Before conducting a definitive RCT, a better understanding of immigrants on the adherence issue is required. A qualitative focus group study exploring their experiences and needs is planned.

Our study has potential limitations, and our results should be interpreted cautiously. The population was small, we had no control group, and we used self-reported questionnaires. The study may suffer from recall bias and social desirability bias. Adherence was mainly based on the MARS questionnaire and should be supplemented with additional adherence measures in the planned RCT. In agreement with the MRC’s recommendations [23], the medication list and person-centered adherence interventions were tested before the planned RCTs.

## Conclusion

In this feasibility study, we have tested LIMM tools and used additional tools to create a process for the interventions that will be investigated in upcoming RCTs. We identified areas that could be changed and improved before future studies; specifically, we encountered recruitment and retention issues that could be resolved by conducting the interventions in two separate studies. Furthermore, to ensure a correct medication list at discharge, we recommend focusing more on achieving a correct medication list in the discharge summary. In summary, we identified several issues related to medication use and adherence that could be solved or improved, and the person-centred adherence intervention was feasible and appropriate. However, the intervention was resource-intensive and needs to be optimised in the RCTs.

### Supplementary Information

Below is the link to the electronic supplementary material.

Supplementary file1 (DOCX 42 KB)
