# Supplementary material for: Intervention for a correct medication list and medication use in older adults: a non-randomised feasibility study among inpatients and residents during care transitions
Source: Int J Clin Pharm. 2024 Feb 10;46(3):639–47. doi: 10.1007/s11096-024-01702-4 (PMC11133128; doi:10.1007/s11096-024-01702-4)
Supplement: Supplementary file 1 — Supplementary file1 (DOCX 42 KB) [file 11096_2024_1702_MOESM1_ESM.docx]

**Intervention for a correct medication list and medication use in older adults – a non-randomised feasibility study among inpatients and residents during care transitions**

Ahmed Al Musawi, Lina Hellström, Malin Axelsson, Patrik Midlöv, Margareta Rämgård, Yuanji Cheng, Tommy Eriksson

Corresponding author and affiliation

Ahmed Al Musawi

Ahmed.Almusawi@mau.se

Department of Biomedical Science and Biofilm – Research Center for Biointerfaces, Faculty of Health and Society, Malmö University, Malmö, Sweden

**Table S1.** Interview guide for the development of the participant’s medication narrative

Would you like to tell us how you started receiving medications?

Tell us about your medications.

What is crucial for you regarding your medication treatment?

Have you considered your medications and their connection to diseases, side effects, etc.? Tell us.

How do you feel about using medication? Some people may feel anxious or have other fears, while others primarily experience them as something positive, as a sense of security.

What do you need to feel safe/even safer with your medication treatment?

- something that needs to be changed

- something that needs to be explained or clarified

- other

How does taking medication affect your daily life?

How does remembering to take medications work for you?

**Table S2.** Structured form for the participant’s health plan

Today, we talked about

My goal setting

- This is what I would like to be able to do or feel – both in the short and long term.

- This is what I am going to do.

- This is what I must do to reach my goals, how and when I will do it, and how I plan to proceed.

My resources

- These are my abilities, qualities, and conditions as help.

Support I need

- I need this support; who can help me and when? It could, for example, be support from a pharmacist, care providers, relatives, etc.

Follow-up, coming meetings

**Table S3.** Description of each participant’s medications during the correct medication list intervention and reasons for not correcting the discrepancies in the Discharge-ML. The column ‘Medications and dosage strength’ represents all medications from different sources. A hyphen indicates the absence of medication in the respective source. Light grey cells indicate discrepancies between the agreed Correct-ML and the participant’s use during the hospital stay. Dark grey cells indicate a discrepancy in the Discharge-ML.

| Patient ID. | Medications and dosage strength | Daily dose according to different sources | | | | | | Comments on medication discrepancies |
| --- | --- | --- | --- | --- | --- | --- | --- | --- |
|  |  | Information sources for a Correct-ML | | | Correct-ML^d^ | Discharge-ML^e^ | Pat-use (follow-up) |  |
|  |  | EHR-ML^a^ | N-ML^b^ | Pat-use^c^ (at home) |  |  |  |  |
| 1 | Omeprazole 20 mg | 1x1 | 1x1 | 1x1 prn^f^ | 1x1 prn | 1x1, 10 tablets/day | 1x1 prn | No medications had deliberately been added or deleted during the hospital stay. Based on the agreement on the Correct-ML, we concluded that the patient had two commission errors and three omission errors during the hospital stay. Since the agreement was established too late and the physician had no time to correct the Discharge-ML, this list also had five medication discrepancies. Based on the discussion with the pharmacist, the patient was aware of the discrepancies in the Discharge-ML. |
|  | Candesartan 8 mg | 2x1 | 2x1 | 2x1 | 2x1 | 2x1 | 2x1 |  |
|  | Oxycodone 5 mg | 1 prn | - | - | - | 1 prn | - |  |
|  | Paracetamol 500 mg | 2x4 | 2x4 | 2x4 | 2x4 | 2x4 | 2x4 |  |
|  | Salbutamol 100 μg/inh. | 1x2 | 1–2 prn | 1–2 prn | 1–2 prn | 1x2 | 1–2 prn |  |
|  | Ethyl morphine cough syrup | 5 ml prn | - | - | - | 5 ml prn | - |  |
|  | Felodipine 2.5 mg | - | 1x1 | 1x1 | 1x1 | - | 1x1 |  |
|  | Zopiclone 7.5 mg | - | 1x1 | 1x1 | 1x1 | - | 1x1 |  |
|  | Budesonide/Formoterol 320/9 μg/inh. | - | 1x2 | 1x2 | 1x2 | - | 1x2 |  |
| 2 | Rivaroxaban 20 mg | 1x1 | 1x1 | 1x1 | 1x1 | 1x1 | 1x1 | Three medications had deliberately been added as new medications.  Agreement on Correct-ML: one omission error during the hospital stay.  The physician missed correcting the Discharge-ML, and one medication discrepancy remained. The patient was aware of the discrepancies in the Discharge-ML. |
|  | Macrogol 3350 | 1x1 | - | - | 1x1 | 1x1 | 1x1 |  |
|  | Amiloride/Hydrochloro-thiazide 2.5/25 mg | 1x2 | 1x2 | 1x2 | 1x2 | 1x2 | 1x2 |  |
|  | Bisoprolol 5 mg | 1x2 | 1x2 | 1x2 | 1x2 | 1x2 | 1x2 |  |
|  | Amlodipine 5 mg | 1x1 | 1x1 | 1x1 | 1x1 | 1x1 | 1x1 |  |
|  | Losartan 100 mg | 1x1 | 1x1 | 1x1 | 1x1 | 1x1 | 1x1 |  |
|  | Oxycodone E.R. 5 mg | 1x2 | - | - | 1x2 | 1x2 | 1x2 |  |
|  | Paracetamol 500 mg | 2x4 | 2x3 | 2x4 | 2x4 | 2x4 | 2x4 |  |
|  | Beclometasone dipropionate, formoterol fumarate dihydrate, glycopyrronium bromide87/5/9 μg | 2x2 | 2x2 | 2x2 | 2x2 | 2x2 | 2x2 |  |
|  | Bimatorpost/Timolol 0.3/5 mg/ml | 1 drp^g^ | 1 drp | 1 drp | 1 drp | 1 drp | 1 drp |  |
|  | Sodium pico sulphate drp. | 10 drp | - | - | 10 drp | 10 drp | 10 drp |  |
|  | Oxycodone 5 mg | 1 prn | - | - | 1 prn | 1 prn | 1 prn |  |
|  | Formoterol fumarate 9 μg | 1 prn | 1 prn | 1 prn | 1 prn | 1 prn | 1 prn |  |
|  | Ipratropium 500 μg/ml | - | 1x2 | 1x2 | 1x2 | - | 1x2 |  |
|  | Paracetamol/Codeine 500/30 mg | - | - | 1-2 prn | - | - | - |  |
| 3 | Omeprazole 20 mg | 1x1 | 1x1 | 1x1 | 1x1 | 1x1 | 1x1 | No medications had deliberately been added or deleted during the hospital stay.  Agreement on Correct-ML: two commission errors and one omission error during the hospital stay.  The physician missed correcting the Discharge-ML, and three medication discrepancies remained. The patient was aware of the discrepancies in the Discharge-ML. |
|  | Macrogol 3350 | 1x1 | 1x1 | 1x1 | 1x1 | 1x1 | 1x1 |  |
|  | Cholecalciferol 800 IE | 1x1 | 1x1 | - | - | 1x1 | - |  |
|  | Felodipine 2.5 mg | 1x1 | 1x1 | 1x1 | 1x1 | 1x1 | 1x1 |  |
|  | Felodipine 5 mg | 1x1 | 1x1 | 1x1 | 1x1 | 1x1 | 1x1 |  |
|  | Paracetamol 500 mg | 2x4 | 2x4 | prn | prn | 2x4 | prn |  |
|  | Olodaterol/tiotropium 2.5/2.5 μg/inh. | 2x1 | 2x1 | 2x1 | 2x1 | 2x1 | 2x1 |  |
|  | Oxycodone 5 mg | 1 prn | 1 prn | - | - | 1 prn | - |  |
|  | Salbutamol 200 μg/inh. | 1 prn | 1 prn | 1 prn | 1 prn | 1 prn | 1 prn |  |
|  | Acetylcysteine 200 mg | 1 prn | 1x2–3 | 1x1 | 1x1 | 1 prn | 1x1 |  |
|  | Calcium/cholecalciferol 500/800 IE | - | 1x1 | 1x1 | 1x1 | - | 1x1 |  |
| 4 | Aspirin 75 mg | 1x1 | 1x1 | 1x1 | 1x1 | 1x1 | 1x1 | No medications had deliberately been added or deleted during the hospital stay.  Agreement on Correct-ML: five commission errors and seven omission errors during the hospital stay.  The physician missed correcting three medication discrepancies in the Discharge-ML, and three medication discrepancies remained. The patient was aware of the discrepancies in the Discharge-ML. |
|  | Bisoprolol 2.5 mg | 1x1 | 1x1 | 1x1 | 1x1 | 1x1 | 1x1 |  |
|  | Alfuzosin 10 mg | 1x1 | 1x1 | - | - | - | - |  |
|  | Finasteride 5 mg | 1x1 | - | - | - | - | - |  |
|  | Prednisolone 10 mg | 1x1 | - | 1x2 | 1x2 | 1x1 | 2.5/day |  |
|  | Sertraline 100 mg | 1x1 | 1x1 | 1x1 | 1x1 | 1x1 | 1x1 |  |
|  | Fluticasone furoate/Vilanterol 184/22 μg/inh. | 1x1 | 1x1 | - | - | - | - |  |
|  | Hydrocortisone ophthalmic ointment 0.5% | 1x3 | 1x3 | - | - | - | - |  |
|  | Iron solution injection 100 mg/ml | 1x3 a week | 1x3 a week | - | - | - | - |  |
|  | Nystatin 100 000 IE/ml | 1 ml prn | 1x4 | 1x4 prn | 1x4 prn | Actp^h^ | 1x4 prn |  |
|  | Furosemide 40 mg | 1 prn | 1x1 | 1x1 | 1x1 | 1x1 | 1x2 |  |
|  | Salbutamol 0.2 mg/inh. | 1 prn | 1 prn | 1 prn | 1 prn | 1 prn | 1 prn |  |
|  | Omeprazole 20 mg | - | 1x1 | 1x1 | 1x1 | 1x1 | 1x1 |  |
|  | Fluticasone furoate/Umeclidinium bromide/Vilanterol 92/55/22 μg/inh. | - | 1x1 | 1x1 | 1x1 | 1x1 | 1x1 |  |
|  | Montelukast 10 mg | - | 1x1 | 1x1 | 1x1 | 1x1 | 1x1 |  |
|  | Paracetamol 1 g | - | 1x3 | 1x3 | 1x3 | - | 1x3 |  |
|  | Candesartan 8 mg | - | 1x1 | 1x1 | 1x1 | 1x1 | 1x1 |  |
|  | Fexofenadine 180 mg | - | 1x1 | 1x1 | 1x1 | - | 1x1 |  |
|  | Cyanocobalamin 1 mg | - | 1x1 | - | - | - | - |  |
|  | Folic acid 1 mg | - | 1x1 | - | - | - | - |  |
|  | Calcium/cholecalciferol 500/800 IE | - | 1x1 | 1x1 | 1x1 | - | 1x1 |  |
| 5 | Apixaban 5 mg | 1x2 | 1x2 | 1x2 | 1x2 | 1x2 | 1x2 | No changes that should be corrected. |
|  | Omeprazole 20 mg | 1x1 | 1x1 | 1x1 | 1x1 | 1x1 | 1x1 |  |
|  | Folic acid 1 mg | 1x1 | 1x1 | 1x1 | 1x1 | 1x1 | 1x1 |  |
|  | Furosemide 40 mg | 1x2 | 1x2 | 1x2 | 1x2 | 1x2 | 1x2 |  |
|  | Eplerenone 25 mg | 1x1 | 1x1 | 1x1 | 1x1 | 1x1 | 1x1 |  |
|  | Metoprolol 50 mg | 1x1 | 1x1 | 1x1 | 1x1 | 1x1 | 1x1 |  |
|  | Magnesium 250 mg | 1x1 | 1x1 | 1x1 | 1x1 | 1x1 | 1x1 |  |
|  | Salbutamol 200 μg | 1x2 | 1x2 | 1x2 | 1x2 | 1x2 | 1x2 |  |
|  | Budesonide/Formoterol 320/9 μg/inh. | 1x2 | 1x2 | 1x2 | 1x2 | 1x2 | 1x2 |  |
|  | Potassium chloride 750 | Prn | Prn | Prn | Prn | - | - |  |
| 6 | Metformin 500 mg | 1x2 | 1x2 | 1x2 | 1x2 | 1x2 | 1x2 | No medications had deliberately been added or deleted during the hospital stay.  Agreement on Correct-ML: one commission error and one omission error during the hospital stay.  The physician missed correcting the Discharge-ML, and two medication discrepancies remained. The patient was aware of the discrepancies in the Discharge-ML. |
|  | Cyanocobalamin 1 mg | 1x1 | 1x1 | 1x1 | 1x1 | 1x1 | 1x1 |  |
|  | Metoprolol 50 mg | 1x1 | 1x1 | 1x1 | 1x1 | 1x1 | 1x1 |  |
|  | Paracetamol 500 mg | 2x3 | 2x3 | - | - | 2x3 | - |  |
|  | Zopiclone 7.5 mg | 1x1 | 1x1 | 1x1 | 1x1 | 1x1 | 1x1 |  |
|  | Escitalopram 20 mg | 1x1 | 1x1 | 1x1 | 1x1 | 1x1 | 1x1 |  |
|  | Acetylcysteine 200 mg | 1 prn | 1 prn | 1 prn | 1 prn | 1 prn | 1 prn |  |
|  | Propranolol 40 mg | - | 1 prn | 1 prn | 1 prn | - | 1 prn |  |
| 7 | Aspirin 75 mg | 1x1 | 1x1 | 1x1 | 1x1 | 1x1 | 1x1 | No medications had deliberately been added or deleted during the hospital stay.  Agreement on the Correct-ML: one omission error during the hospital stay.  The physician corrected the medication discrepancy in the Discharge-ML. |
|  | Furosemide 40 mg | 1x1 | 1x1 | 1x1 | 1x1 | 1x1 | 1x1 |  |
|  | Metoprolol 25 mg | 2x1 | 2x1 | 1x2 | 2x1 | 2x1 | 2x1 |  |
|  | Amlodipine 5 mg | 1x2 | 1x2 | 1x2 | 1x2 | 1x2 | 1x2 |  |
|  | Atorvastatin 40 mg | 1x1 | 1x1 | 1x1 | 1x1 | 1x1 | 1x1 |  |
|  | Paracetamol 500 mg | 2x3 | 2x3 | 2x3 | 2x3 | 2x3 | 2x3 |  |
|  | Ramipril 2.5 mg | - | 0.5x1 | 1x1 | 0.5x1 | 0.5x1 | 0.5x1 |  |

^a^The medication list in the electronic health record, ^b^The national medication list, ^c^Patient use, ^d^Correct medication list, ^e^Discharge medication list, ^f^As needed, ^g^Drops, ^h^According to prescription/agreement

We followed and documented each participant’s processes to evaluate the intervention progression. Nine participants had several goals related to needing more knowledge about the medication. Six participants expressed a need to know whether there were interactions between their medication. One participant wanted to know whether there was a connection between the medications and the nightmares he was suffering from. One participant requested help to remember medication intake. One participant wanted to know which medication needed to be taken, and three reported a need to reduce the number of medications. Further, one participant requested more knowledge about dementia and its available medication, and another wanted to know more about hypothyroidism and suitable food, reflux disease, and prescribed medications. Table S4 describes the intervention’s process and progression for one hospital participant and one residential participant.

**Table S4.** Examples of the intervention process for one hospital patient and one residential participant.

| Hospital patient (No. 7) | Residential participant (No. 8) |
| --- | --- |
| As presented in Table 4, this participant was classified as ambivalent and with high medication adherence. The participant had problematic health literacy and expressed an interest in learning more about the medications but was concerned about their possible interactions with each other. However, the participant had no plans to stop taking them. In the (first) follow-up interview (of five in total), we discussed each medication, its use, and how it may interact with other medications. We set goals for the follow-up meetings and target dates to achieve goals. The first issue was about the analgesics and anti-inflammatory medications. The participant had been using those for some time despite having heart and blood pressure problems and despite bruising on the skin being a possible side effect. The participant also expressed concern about the generic substitution of medications in pharmacies. We discussed the background for changes and pharmacy regulations. The participant expressed satisfaction in having a pharmacist to talk to about the medications. At study termination, the participant was classified as accepting in the BMQ-S and still had high adherence, with a slightly higher score on the MARS-5 than at the beginning. | As presented in Table 4, this participant was classified as ambivalent and with low adherence. The participant had problematic health literacy and did not believe in medications. The participant did not know what medications were prescribed and why they should be taken, and it was not easy to establish a correct medication list. We set a goal to visit the physician to establish a correct medication list. According to the physician, four medications did not need to be used. The essential medications were amlodipine and losartan/hydrochlorothiazide, atorvastatin, acetylsalicylic acid, and omeprazole.  Together, we set a goal for the upcoming follow-up meetings to discuss what these medications are and why it is essential to take them as prescribed. The participant became more interested in her medications and began taking the blood pressure and cholesterol medications. As presented in Table 4, the participant improved total MARS scores and decreased concern points in BMQ-S but still counted as ambivalent. |
